# A Pilot Study Quantifying Center of Mass Trajectory during Dynamic Balance Tasks Using an HTC Vive Tracker Fixed to the Pelvis

**DOI:** 10.3390/s21238034

**Published:** 2021-12-01

**Authors:** Susanne M. van der Veen, James S. Thomas

**Affiliations:** Department of Physical Therapy, Virginia Commonwealth University, Richmond, VA 23298, USA; jtjomas32@vcu.edu

**Keywords:** center of mass, balance, berg balance scale, virtual reality

## Abstract

Fall rates are increasing among the aging population and even higher falls rates have been reported in populations with neurological impairments. The Berg Balance Scale is often used to assess balance in older adults and has been validated for use in people with stroke, traumatic brain injury, and Parkinson’s disease. While the Berg Balance Scale (BBS) has been found to be predictive of the length of rehabilitation stay following stroke, a recent review concluded the BBS lacked predictive validity for fall risk. Conversely, sophisticated measures assessing center of mass (COM) displacement have shown to be predictive of falls risk. However, calculating COM displacement is difficult to measure outside a laboratory. Accordingly, we sought to validate COM displacement measurements derived from an HTC Vive tracker secured to the pelvis by comparing it to COM derived from ‘gold’ standard laboratory-based full-body motion capture. Results showed that RMS between the COM calculated from HTC Vive tracker and full body motion capture agree with an average error rate of 2.1 ± 2.6 cm. Therefore, we conclude measurement of COM displacement using an HTC Vive tracker placed on the pelvis is reasonably representative of laboratory-based measurement of COM displacement.

## 1. Introduction

Falls are a great cost to the society. Approximately $50 billion is spent on non-fatal falls and another $754 million on 28,486 fatal falls yearly [1]. Most falls happen in elderly populations, and even more in populations with balance deffecits associated with stroke [2,3], traumatic brain injury (TBI), or Parkinson’s disease (PD) [4]. The Berg Balance Scale (BBS) is often used to assess balance in clinic and consists of 14 tasks (Table 1) that a patient completes, and therapist scores on a 0–4-point scale. A 0 is scored if the patient is unable to do the task at all and 4 if they can complete the task perfectly. The max score is 56 and a score of less than 45 has been identified as a cut point for increased falls risk [5,6]. Some of the benefits of the BBS are that it is brief, easy to administer, is publicly available at no cost, and requires no specialized training for the assessor. The BBS is a well-established and validated [7,8,9] clinical tool for measuring balance deficits and has been validated for balance assessment in a wide range of populations—including stroke, Parkinson’s disease, and TBI [7,8,9,10,11,12,13,14]. The BBS has been found to be predictive of length of stay, discharge destination, motor ability and disability level post-stroke and low fall risk [15]. However, the BSS does not have strong predictive value of high falls risk [8,11].

In recent research, the use of higher order derivatives (i.e., angular velocity and linear acceleration) of COM displacement to predict falls and postural stability [16,17,18] has been growing, and could better quantify falls risk [16]. High levels of accuracy, specificity, and sensitivity have been achieved in falls risk prediction based on COM displacement [16,18,19]. Thus, COM is often used as a measure of falls risk and has previously been validated using inertial sensors [16]. However, the data extraction, processing and interpretation can be quite challenging, as different activities require specific analysis. To make COM data collection and the interpretation readily available for clinicians, we used a subset of the standardized and validated tasks of the BBS in our novel virtual reality (VR) based BBS (i.e., VR-BBS), and plan to incorporate the VR-BBS with task specific falls risk assessment algorithms. First, however, the used of positional data of the HTC Vive tracker a proxy of COM displacement must be established. This would extend our previous work on validation of an HTC Vive tracker for positional tracking [20].

Therefore, we have instrumented participants with an HTC Vive tracker (HTC, Inc. Taiwan) on their pelvis, as a proxy for COM. In previous studies, the HTC Vive tracker has shown to be accurate in tracking joint position and rotation data [20] and center of pressure (COP) displacement [21], however validation of COM trajectory with the HTC Vive tracker has not been studied. The purpose of this study was to determine if the VR-BBS toolkit can provide valid COM displacement data based on the HTV Vive tracker attached to the pelvis consistent with COM displacement using a motion capture laboratory during a subset of original BBS tasks.

## 2. Materials and Methods

HTC Vive position data of the pelvis and the head mounted display (HMD) was simultaneously collected with full body kinematics during the VR-BBS where participants completed 12 of the 14 BBS tasks (See bolded items Table 1). Tassk 4 and 5 were omitted for safety reasons; however, the chair used in item 2 and 3 was represented in the VR environment using an additional HTC Vive tracker.

### 2.1. Motion Capture Systems

#### 2.1.1. HTC Vive System

Two HTC Vive trackers (HTC America, Inc., Seattle, WA, USA) were used to track the position of the pelvis and chair in the Vive VR system. Four fixed infrared laser emitter second generation “Lighthouses” (HTC Vive Lighthouses, Valve Washington, WA, USA) were used to register position and orientation of these trackers.

Custom software developed in the Unity game engine, (version 2018.2.6 f1 Unity Technologies, San Francisco, CA, USA), created the virtual environment and system to collect the six-degrees of freedom (DOF) kinematic data from the Vive trackers with a sampling frequency of 100 Hz.

#### 2.1.2. Vicon System

An 18-camera 3D optoelectric kinematic system, consisting of 12 Vicon Vero 2.2 and 6 Vantage 5 cameras (Vicon Motion Systems Ltd., Oxford, UK) tracked the 6-DOF position and orientation of 16 custom 3D-printed light-reflective marker were attached to hands, lower arms, upper arms, feet, shanks, thighs, lumbar, thorax, sacrum, and head segments of the participants. The 6-DOF kinematic data from the body segments were collected at a sampling frequency of 100 Hz (with a spatial resolution of 0.1 mm) and Euler Angles derived using Motion Monitor software (Innovative Sports Training, Chicago, IL, USA).

### 2.2. Immersive Virtual Reality Envrionement

**Figure 1:**
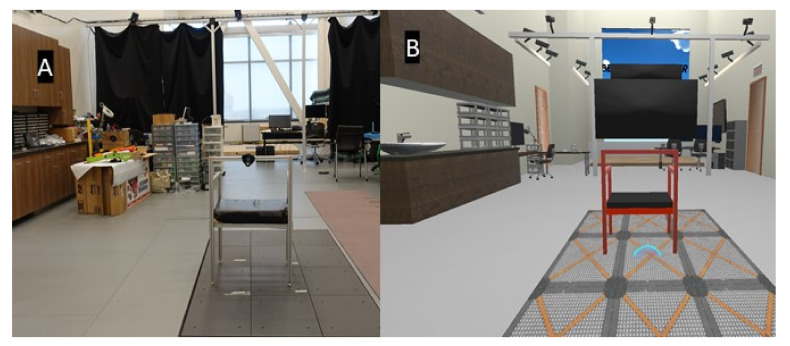
Representation of the environment in (**A**) real world and, (**B**) Virtual Reality.

**Figure 2:**
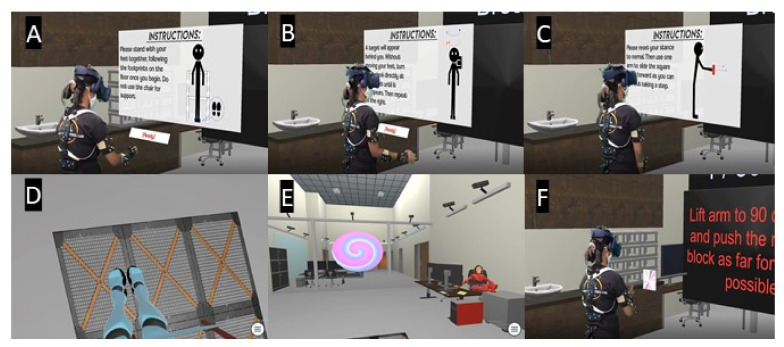
A representation of Virtual Reality Berg-Balance Scale ((VR-BBS) **A**,**D**) Narrow stance, the instructions read: Please stand with your feet together, following the footprints on the flow once you begin. Do not use the chair for support. ((task 4 in VR and task 7 in the standard BBS, see Table 1), **B**,**E**) Turning to look behind over left and right shoulder while standing. The instructions read: A target will appear behind you. Without moving your feet, turn to look directly at the target to the left until it disappears. Then repeat to the right. ((VR-BBS task 6 standard BBS task 10) and **C**,**F**) Reaching forward with outstretched arm while standing. The instructions read: Please reset your stance to normal. Then use one arm to slide the square as far forward as you can without taking a step. ((VR-BBS task 5 standard BBS task 9).

A custom virtual Berg Balance scale, the *VR-BBS*, was created using Unity software and provided a representation of our lab space, including a physical chair that was visualized within the VR environment using an HTC Vive tracker (See Figure 1). The game requires players to carry out a subset of tasks in the BBS (see Table 1). Some of these tasks were modified for safety reasons. Specifically, tasks 4 and 5 were eliminated to reduce falls risk, and task 12 was modified to so a real-world step stool would not be needed. Figure 2 illustrates a participant within the VR-BBS (using green screen technologies) to provide a subject’s perspective of task instructions and tasks. The HTC Vive system (HTC America, Inc., Seattle, WA, USA) was used to allow physical movement of the virtual avatar within the virtual environment. The participants were immersed using a wired HMD (470 g) that uses an organic light-emitting diode display and provides a resolution of 1080 × 1200 per eye, with a refresh rate of 90 Hz, and a field of view of 110°.

### 2.3. Measurement Set Up

A 4 × 8 m laboratory was fitted with four HTC Vive lighthouses and 18 Vicon cameras to cover adequate data collection volume for unconstrained game play. The axes of the world reference frame for the Vicon system was such that positive z faces upward, positive x faces forward, and positive y faces leftward relative to the position of the subject. Thus, flexion of the spine would result in a clockwise rotation about the y-axis and a forward displacement along the x-axis. Twisting of the trunk would result in a rotation about the z-axis with minimal displacement along the x- or y-axes.

### 2.4. Participants

Thirteen healthy adults between the age of 18 and 35 were recruited for this study. The exclusion criteria included (1) vision problems or, (2), any orthopedic or neurological impairment that would prevent participants from executing tasks that require moderate amounts of trunk flexion. The Institutional Review Board of Virginia Commonwealth University, approved the study protocol (HM20014688) and all participants signed an informed consent form. In compensation for their time, participants received an Ohio University Motor Control Lab t-shirt.

The participants were asked to wear shorts, a shirt and gym shoes provided by the laboratory. Vicon light reflective trackers were attached to custom designed 3D printed plates to create marker clusters for measuring 6-DOF. The pelvis plate was designed to allow the attachment of HTC Vive trackers in the center of the marker clusters, thus co-locating the two sensors [20]. The 3D printed components were attached to the thorax and sacrum using elastic straps.

### 2.5. Data Collection

Displacement of whole-body COM time series were derived relative to the global coordinate system using the MotionMonitor system (Chicago, IL, USA). Whole body COM was also approximated from pelvis position with the HTC Vive tracker using Unity software. The difference is coordinate systems were corrected by translating the data from the HTC Vive trackers to conform to the world axes of the Vicon system. The time-series Euler angle data and position data from the Vicon cluster and the HTC Vive trackers were exported from The Motion Monitor software. These time series data were imported into MATLAB and processed using custom programs. All data were smoothed using a 40-point Savitzky–Golay filter and DC offset removed. The two data sets were temporally aligned based on known events (initial start of game output from Unity game engine).

### 2.6. Outcomes

Root means square (RMS) errors were computed for the time series data and the mean difference) between COM and HTC Vive tracker area in X (anteroposterior) and Y (mediolateral) direction was calculated per standing balance VR-BBS measure (achieved by subtracting the minimum of the time series from the maximum of the time series).

### 2.7. Statistical Analysis

RMS were computed on the time-series data from the Vive and Vicon positional and orientation data streams for the 12 BBS tasks separately. In total 12 tasks for 13 participants would lead to 156 calculated RMS trials, however, for 29 trials spread over 10 of the participants were not analyzed due to missing data. That is, either COM (*n* = 21) or HTC Vive tracker (*n* = 8) time series were unavailable (numbers are represented in Table 2).

ANOVA measures were used to test for significant differences between COM area derived from HTC Vive and Vicon for each task in both X and Y directions. A Bayesian repeated measures ANOVA was calculated across VR-BBS tasks for X and Y direction with open-source JASP software using default priors (JASP team, 2017). Bayes Factor (BF_10_) were reported expressing the probability of the similarity between COM and HTC Vive tracker data and assessed using Lee and Wagemakers classification scheme [22], where values smaller than 1 represent increasing favor towards the null hypothesis.

## 3. Results

Thirteen (5 Male, Age in years Mean ± STD 27 ± 8) adults were recruited for this study, four of them had reported one or more falls in the past 12 months (these falls were all sport related), but none of the participants reported to have fall-related self-efficacy (falls efficacy scores ranged from 0–21, scores above 70 has been indicative for fear of falling [23,24]). This measure does not represent fear of falling and may not be representative in the healthy young participants taking part in this validation study.

### 3.1. RMS

**Figure 3:**
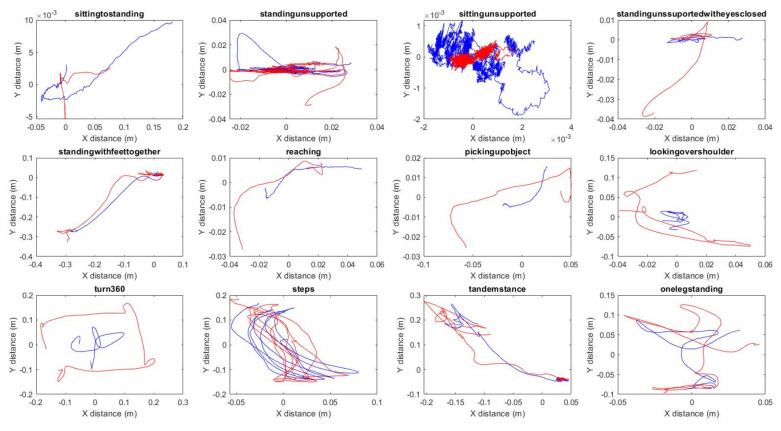
Task data of one of the subjects completing the VR-BBS, the blue line represents the COM and the red line the HTC Vive tracker trajectory.

Positional RMS error, averaged across participants and tasks to be mm in 2.6 ± 2.8 cm in X, 2.4 ± 3.0 cm in Y, and 1.2 ± 1.6 cm in Z direction. However, an average RMS of 2 cm represents close tracking of the COM by the HTC Vive tracker, as you can see in Table 2 and Figure 3, the COM measurements derived by the two systems shows greater deviations in the more dynamic tasks such as picking up an object, looking over the shoulder, and turning 360°.

### 3.2. Displacement

The COM and HTC Vive tracker area were moderately similar (for the X BF_10_ = 0.0326 and Y BF_10_ = 0.308) over the five standing tasks of the VR-BBS; standing unsupported, standing with eyes closed, standing with a narrow base of support, tandem stance, and one leg stance, see Table 3 for values per task.

## 4. Discussion

This paper aimed to establish the agreement between COM displacement measured using a VR tracking system (i.e., HTC Vive tracker) and a 3D optoelectric kinematic system (the MotionMonitor, Chicago, IL, USA) during a session of VR-BBS. As a traditional method of motion tracking, Vicon has established an accuracy of 0.1 mm and 0.1 within a calibrated volume space. Use of three-dimensional kinematic measures has shown to be a good estimator of body COM in previous literature [25]. This analysis shows that a HTC Vive tracker placed on the pelvis agree with validated COM displacement with an average RMS error of 2.1 ± 2.6 cm.

In this study, the distance traveled in X and Y direction during the standing tasks of the BBS were not significantly different between the COM based on the HTC Vive tracker (direction X BF_10_ = 0.0326, and Y BF_10_ = 0.308) and 3D kinematics. However, the different standing balance tasks show greater deviations in harder tasks (e.g., narrow base of support and tandem stance, than easier ones; standing unsupported and standing with eyes closed). One-leg standing is generally assumed to be harder than standing with a smaller base of support but had smaller differences in COM derived with the two methods. HTC Vive tracker deviations, this could be explained by the foot-sized base of support in this task, which likely limited the COM displacement to maintain postural stability.

Although the agreement between COM and the HTC Vive tracking overall was in agreement with an error of 2.1 ± 2.6 cm, certain tasks had greater differences than others. Specifically, the 360° turn (X 10.44 ± 1.46, Y 9.12 ± 1.36, Z 0.66 ± 0.17), picking up an object (X 6.43 ± 0.60, Y 1.81 ± 1.36, Z 4.14 ± 2.90), and looking over the shoulder (X 2.86 ± 0.98, Y 7.97 ± 2.72, Z 0.54 ± 0.18) had greater errors. These dynamic tasks require larger movements of the limbs and trunk segments and inherent kinematic redundancies allow them to be completed with a near infinite variety of limb excursions. As we are approximating COM based on a single tracking point, it is logical that the differences between COM derived with the HTC Vive tracker and the 3D optoelectric kinematic system will be larger. Although we reported RMS errors as big as 12 cm, the trajectories of the pelvis are merely magnifications of displacement of COM. Finally, if these displacements are accounted for in higher order derivatives for fall risk estimation algorithms, the Vive derived COM displacements may allow for greater sensitivity in identifying falls risk in patient population.

This study is the first to assess COM tracking capabilities of the HTC Vive tracker; however, positional tracking of the sacrum [20] and center of pressure [21] have been validated before. Both studies reported high agreement of the compared motion data. Liang et al. showed that the discriminative power between the four conditions they had; standing unsupported with eyes open and closed and standing with narrow base of support with eyes open and closed. Three of these four conditions are very similar to VR-BBS conditions, but they reported smaller deviations of the HTC Vive tracker in standing with eyes open and closed that we did. This is probably caused by specific foot-placement in the standing unsupported with eyes open and closed (15 cm apart under a 25° angle) [21] and the measurement time of 30 s. In this study, the original BBS protocol was more closely followed, without instructions about foot width, and with two minutes of standing with eyes open. The eyes-closed condition was 30 s compared to 10 s in the original BBS. This increase is to allow for higher order analyses of COM displacement. It is likely that people were more balanced in their neutral stance than the very specific stance the study by Liang et al. [21] required.

While position, angular velocity, and linear acceleration of the pelvis has been used widely to assess balance and predict fall risk [16], VR-based tracker data has not been examined in the literature. In a review by Howcroft et al. [16] 65% of the studies placed the tracker (accelerometer or gyroscope) on the lower back, pelvis or sacrum to approximate COM [26,27,28]. Many studies used tasks similar to the BBS such as sit-to-stand [29], standing unsupported [26,30,31], tandem stance, standing with narrow base of support [32], alternate stepping [33,34,35], and one leg stance [36]. The accuracy, specificity, and sensitivity in these studies ranged 65.5–97, 35–98.5, and 55–99 respectively, which is very promising as optimal variables and assessment can be identified.

Several limitations are worth considering. First, participants were healthy young individuals and demonstrated a relatively stable posture. For example, elderly people tend to rely on a pelvic control strategy instead of a youth-preferred ankle control strategy. Therefore, a different correlation may exist between COM displacement measured using motion capture versus the HTC Vive tracker. Furthermore, studies are warranted to help to establish the feasibility of this system with various populations. Secondly, data of 10 of the participants was not complete due to occlusion of one of the body segments, preventing full body COM to be calculated, or HTC Vive tracker going in to sleep mode when they had been stationary for too long. This loss of data can be prevented in the future by making sure marker placement and camera positioning are optimized, and limiting the time participants had to wait in-between tasks.

## 5. Conclusions

We designed a VR-BBS equipped with a Vive tracker-based system as a simple and easy-to-use tool for evaluating postural stability. The results showed a good convergent validity in comparison to the COM data from a 3D kinematic camera system. This system has the potential to be a screening tool that has the advantages of low cost, high mobility, and simple setup and can be integrated into simulated conditions created by a VR system to evaluate body sway in virtual environments. Further studies in various populations should be conducted to establish the feasibility of wider application.

## Figures and Tables

**Table 1:** The 14 tasks in the Berg Balance Scale.

Task Number	Description
1	Sitting to standing
2	Standing unsupported
3	Sitting with back unsupported but feet supported
4	Standing to sitting
5	Transfer from chair to chair
6	Standing unsupported with eyes closed
7	Standing unsupported with narrow base of support
8	Reaching forward with outstretched arm while standing
9	Pick up object from the floor from standing position
10	Turning to look behind over left and right shoulder while standing
11	Turning 360°
12	Placing alternate foot on stool
13	Standing unsupported one foot in front
14	Standing on one leg

**Table 2:** Representing root mean square error between the HTC Vive tracker and the COM in X, Y, and Z direction for the 12 BBS tasks.

	COM X (cm)	COM Y (cm)	COM Z (cm)
Sitting to standing *n* = 13	2.35 ± 1.65	0.27 ± 0.17	2.53 ± 1.36
Standing unsupported *n* = 10	0.34 ± 0.24	0.31 ± 0.17	0.19 ± 0.07
Sitting unsupported *n* = 9	0.14 ± 0.15	0.10 ± 0.05	0.12 ± 0.15
Standing unsupported with eyes closed *n* = 10	0.66 ± 0.68	0.49 ± 0.38	0.29 ± 0.29
Standing with feet together *n* = 10	1.16 ± 0.38	0.94 ± 0.29	0.46 ± 0.11
Reaching *n* = 11	2.28 ± 1.29	1.57 ± 1.06	1.55 ± 0.94
Picking up object *n* = 12	6.43 ± 0.60	1.81 ± 1.36	4.14 ± 2.90
Looking over shoulder *n* = 10	2.86 ± 0.98	7.97 ± 2.72	0.54 ± 0.18
Turn 360 *n* = 8	10.44 ± 1.46	9.12 ± 1.36	0.66 ± 0.17
Alternative stepping *n* = 10	1.83 ± 0.45	4.13 ± 1.60	1.21 ± 0.71
Tandem stance *n* = 7	1.80 ± 0.34	1.70 ± 0.51	0.74 ± 0.23
One leg standing *n* = 12	1.59 ± 0.83	2.06 ± 1.10	0.90 ± 0.48

**Table 3:** Distance of the COM travelled in X- and Y-direction during the subsequent VR-BBS tasks in cm.

	COMX	HTC Vive TrackerX	COMY	HTC Vive TrackerY
Standing unsupported (cm)	3.5 ± 1.0	3.7 ± 0.9	0.2 ± 1.1	2.3 ± 1.4
Standing with eye closed (cm)	2.1 ± 1.2	2.5 ± 1.0	0.9 ± 0.3	1.9 ± 1.6
Narrow base of support (cm)	26.6 ± 3.3	30.3 ± 3.9	25.5 ± 0.063	28.6 ± 7.8
Tandem stance (cm)	19.6 ± 6.2	22.5 ± 2.8	28.3 ± 6.3	31.2 ± 8.9
One leg stance (cm)	8.2 ± 2.7	9.3 ± 4.5	13.9 ± 6.3	15.2 ± 7.7

## Data Availability

Not applicable.
